# Allostatic Load as a Measure of Cumulative Physiological Stress in Cancer: Implications for Prehabilitation in Head and Neck Cancers—A Narrative Review

**DOI:** 10.3390/cancers18111854

**Published:** 2026-06-05

**Authors:** Mariusz Kiszka, Anna Skotny, Magdalena Kanicka, Emilia Burnejko-Jaśkiewicz, Szczepan Barnaś, Piotr Barnaś, Marcin Łaśko, Dorota Kamińska

**Affiliations:** 1 4th Military Hospital, 50-981 Wrocław, Poland; askotny@4wsk.pl (A.S.); mkanicka@4wsk.pl (M.K.); ejaskiewicz@4wsk.pl (E.B.-J.); pbarnas@4wsk.pl (P.B.); dkaminska@4wsk.pl (D.K.); 2 Harvard T.H. Chan School of Public Health—Executive and Continuing Education, Harvard University, Boston, MA 02115, USA; 3 Faculty of Medicine, Wrocław University of Science and Technology, 50-370 Wrocław, Poland

**Keywords:** allostatic load, head and neck cancer, prehabilitation, postoperative complications

## Abstract

Allostatic load (AL) is a multisystemic indicator of the cumulative “wear and tear” on the body caused by chronic stress. In oncology, high AL is associated with a poorer prognosis, a higher number of postoperative complications, and lower treatment tolerance. Patients with head and neck cancer (HNC)—due to frequent smoking, alcohol abuse, low socioeconomic status, and high psychological and functional burden—belong to a group particularly vulnerable to high AL; however, its role in this population remains poorly understood. This narrative review includes publications from 2015 to 2026 from the PubMed/MEDLINE, Embase, and Scopus databases. We analyzed original studies, systematic reviews, and narrative reviews concerning AL in oncology, prehabilitation, and HNC. Additionally, we employed the snowballing method and included studies from key research groups. The results reveal a clear research gap—the lack of direct studies evaluating AL in HNC patients. In other cancers (breast, colorectal, lung), high AL is an independent risk factor for complications, longer hospital stays, and poorer survival. Multimodal prehabilitation (exercise, nutritional, and psychological support) shows potential for reducing AL, but no prospective studies evaluating this effect have been conducted in the HNC population. Assessment of AL may serve as a valuable tool for preoperative risk stratification and monitoring the effects of prehabilitation in patients with head and neck cancer. Prospective cohort and randomized trials are needed to integrate AL into precision medicine for this patient group.

## Simple Summary

In patients with head and neck cancer, the accumulation of stressors (smoking, alcohol, difficult social circumstances, and the psychological burden of the disease) leads to what is known as allostatic load—the physiological “wear and tear” on the body. High levels of this burden are associated with poorer treatment outcomes, a higher number of complications, and shorter survival. This review highlights this underestimated phenomenon and indicates that prehabilitation (multi-stage preparation for surgery: exercise, nutrition, psychological support) can reduce it. The authors call for urgent research on measuring allostatic load in preoperative assessment. This would enable better risk stratification, improved treatment safety, and a reduction in health inequalities, and consequently better treatment outcomes and a higher quality of life for patients.

## 1. Introduction

Allostasis describes the body’s ability to maintain homeostasis under changing conditions. The concept of allostasis and its long-term cost—allostatic load (AL)—was introduced by B. S. McEwen and E. Stellar at the end of the last century [1]. AL represents the cumulative biological burden resulting from repeated or chronic activation of the hypothalamic–pituitary–adrenal (HPA) axis and the sympathetic nervous system, as well as the resulting multi-organ dysregulation. AL integrates biomarkers from various systems: cardiovascular, metabolic, immunological, and neuroendocrine [2]. This provides a comprehensive picture of the stress burden on the entire body, including cortisol, the best-known stress hormone. It can be argued that AL effectively captures physiological ‘wear and tear’ and its potential impact on the development and progression of diseases, including cancer [3].

In recent years, oncology has increasingly focused on AL, which may also serve as a predictive factor in assessing the risk of postoperative complications [4,5]. Chronic stress and the resulting physiological dysregulation promote carcinogenesis through a wide range of mechanisms, such as suppression of the immune response, enhanced angiogenesis, or alteration of the tumor microenvironment. During therapy, high AL negatively affects treatment tolerance and accelerates tumor progression, which translates into poorer survival [3,4,5]. This is confirmed not only by individual studies but also by a meta-analysis [3]. High AL is associated with poorer overall survival and higher cancer-specific mortality, a greater risk of postoperative complications, and a varied response to treatment depending on the type of cancer—e.g., in patients with breast, colorectal, or lung cancer.

Despite the growing role of AL as a predictive factor in oncology in recent years, knowledge regarding its predictive value remains limited for many tumor sites. Head and neck cancers (HNC), the majority of which are head and neck squamous cell carcinomas (HNSCC), serve as an example. HNCs account for approximately 5% of all malignant tumors worldwide [6].

Patients with HNC are characterized by a particularly high risk of elevated AL even in the preoperative period. They typically have classic risk factors, such as smoking and alcohol abuse, low socioeconomic status, social isolation, and significant psychological distress resulting from the tumor’s location and its treatment, which are associated with both functional deficits and a negative impact on physical appearance [6,7,8,9].

These factors may significantly contribute to the accumulation of allostatic load. The lack of dedicated studies assessing the role of AL in this patient group can be considered a significant clinical and research gap.

**Figure 1:**
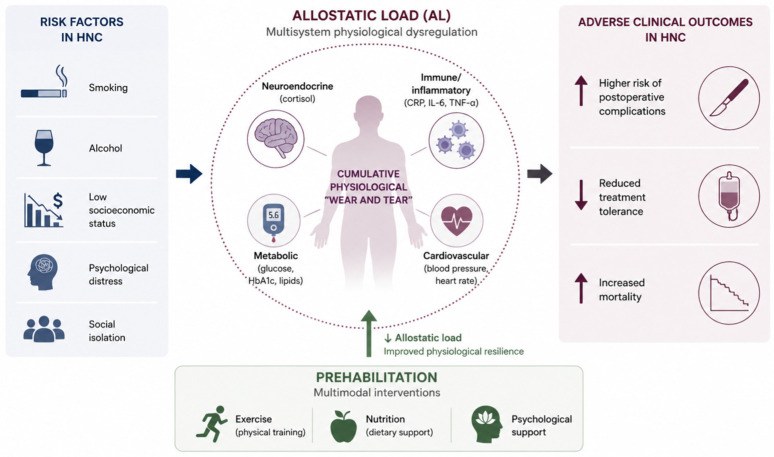
Conceptual framework illustrating the role of allostatic load (AL) in head and neck cancer (HNC).

In the context of preparing patients for surgical treatment, prehabilitation is becoming increasingly important [10]. Its main goal is to optimize physical and mental health prior to surgery—physically, primarily to prevent malnutrition and sarcopenia, and psychologically, to reduce the psychological burden associated with the diagnosis and its consequences. Multimodal prehabilitation may also have a beneficial effect on the immune system. Given the broad spectrum of prehabilitation activities, the measurement of AL appears to be a particularly valuable tool for the precise assessment of intervention effects and the stratification of the risk of postoperative complications [5,11,12]. Consequently, assessing AL in HNC patients prior to surgery may allow for greater personalization of treatment preparation, thereby improving prognosis and quality of life. The conceptual framework linking risk factors, allostatic load, and clinical outcomes in HNC, as well as the potential modifying role of prehabilitation, is presented in Figure 1.

The aim of this narrative review is to discuss the role of AL in oncology, with a particular focus on HNC. The study presents various methods for calculating AL and analyzes its potential as a tool for stratifying the risk of postoperative complications and monitoring the effects of prehabilitation in patients awaiting surgery, thereby filling a significant knowledge gap in this patient population.

## 2. Search Strategy

This narrative review included original research studies, systematic reviews, narrative reviews, and review articles published between 2015 and 2026. The search was conducted in the PubMed/MEDLINE, Embase, and Scopus databases using the following keyword combinations: “allostatic load”; “allostatic load” AND “cancer”; “allostatic load” AND (“head and neck cancer” OR “HNC” OR “HNSCC”); “prehabilitation” AND “cancer”; “allostatic load” AND “prehabilitation”; “allostatic load” AND “postoperative complications”. Particular attention was paid to studies reporting standard biomarkers of allostatic load across the following domains: cardiovascular (e.g., blood pressure, heart rate), anthropometric (e.g., BMI, waist-to-hip ratio), inflammatory/immunological (e.g., CRP, IL-6, TNF-α), metabolic (e.g., glucose, HbA1c), and neuroendocrine (e.g., cortisol) [3,13]. Additional sources included manually searched reference lists of selected articles (snowballing) and works by key research groups specializing in allostatic load in oncology (e.g., Mathew, Obeng-Gyasi, Khalil). The most recent search was conducted in April 2026. Due to the nature of the narrative review, no formal inclusion or exclusion criteria were applied.

Generative artificial intelligence (GenAI) tools were used in the preparation of this manuscript. The DeepL model was used to translate the original text from Polish into English. The ChatGPT model (GPT-5.5, accessed May 2026) was used to prepare Figure 1 (conceptual diagram) in accordance with the authors’ precise instructions. The entire scientific concept, the structure of the paper, the selection of references, the interpretation of results, and the final editing of the text are the sole responsibility of the authors. GenAI tools were not involved in the study design, data analysis, or the formulation of scientific conclusions. The final version of the manuscript has been fully reviewed, substantively revised, and approved by the authors.

## 3. Discussion

### 3.1. Models for Calculating AL

In medicine, assessing a patient’s health status beyond standard laboratory results is usually difficult. Calculating AL is no exception in this regard. Since the introduction of this concept into clinical practice at the end of the last century, several different models for calculating AL have been developed. This clearly indicates that the “golden standard” is still being sought, as each of the methods used has its own advantages and disadvantages [2,3,14].

One of the most commonly used methods for calculating AL, both in scientific research and clinical practice, is the high-risk quartile method [15]. It involves assigning 1 point for each biomarker falling into the high-risk category. The sum of the points obtained constitutes the final measure of AL [16]. Although this is one of the most popular methods, it has significant limitations. The most important of these are the strong dependence of the result on the distribution of biomarker values in the study sample and the inability to compare results across different populations [17]. Even within this method, numerous variants are used—differences concern both the selection of biomarkers and the cut-off points adopted. A growing group of researchers is currently moving toward combining laboratory biomarkers with clinical variables, which may increase the practical utility of AL [17].

Another method used in clinical practice is the clinical cut-off method. In this approach, AL scores are assigned based on widely accepted clinical thresholds, such as blood pressure values defining hypertension, body mass index (BMI) for obesity, HbA1c levels, or C-reactive protein (CRP). It should be emphasized, however, that the thresholds used may vary depending on the criteria adopted and the study population. The main advantage of this method is the relative ease of interpreting results and the ability to compare them across different studies. Nevertheless, this approach underestimates AL in subclinical disorders, as it does not account for deviations that still fall within the normal range [3,18].

The third method, used less frequently than the two described above, is standardization using z-scores, which involves converting the values of each biomarker into normalized values and then summing or averaging them. This method allows for the use of full continuous data and does not require the arbitrary setting of cut-off points. However, it is more computationally demanding, and in the case of biomarkers with a skewed distribution (e.g., CRP), prior logarithmic transformation is recommended [19].

The use of more complex mathematical formulas allows for the development of even more advanced methods for assessing AL. One example is system-weighted indices, in which different physiological systems are assigned varying weights based on their clinical significance. Among modern approaches, it is also worth noting item response theory (IRT), which accounts for the varying “difficulty” and discriminatory power of individual biomarkers, thereby allowing for a more precise estimation of AL levels [20]. Some authors also highlight the need to account for medication usage, as certain medications may mask the actual level of AL [21]. In recent years, machine learning-based approaches have also been tested, which have the potential to better capture the complex interactions between biomarkers [22].

The choice of method for calculating AL significantly affects study results and the ability to compare them across populations. A detailed comparison of the main methods is presented in Table 1. While simpler methods (such as high-risk quartiles and clinical cut-offs) remain widely used due to their practicality, more advanced statistical techniques, including IRT and machine learning, offer greater precision at the cost of increased methodological complexity.

### 3.2. Allostatic Load in Cancer—Current State of Knowledge

The concept of AL originated outside the field of oncology, but in recent years—due in part to advances in diagnostic and therapeutic methods—it has become an essential component of this field. AL reflects the body’s physiological “wear and tear.” From a clinical perspective, this means that AL can be used to assess potential tumor progression or response to treatment, but it can also serve as a good indicator of a patient’s quality of life, which, from the patient’s perspective, represents a critically important outcome measure.

The systematic review conducted by Mathew et al. can be considered one of the first and most important studies to demonstrate a link between AL and the prognosis of cancer patients [3]. The results of this systematic review showed that a one-unit increase in AL was associated with an approximately 9% increased risk of cancer-specific mortality. This finding is significant not only from a statistical standpoint but, above all, from the perspective of routine clinical practice. Additionally, the mini-meta-analysis included the medical histories of over 30,000 patients. These results were confirmed in subsequent cohort studies. High AL was found to be an independent risk factor for poorer overall survival, including in patients with lung, breast, or ovarian cancer [4,23,24].

Furthermore, a study conducted by M. Khalil and T. M. Pawlik provided crucial evidence [25]. In a group of over 40,000 patients who underwent colorectal cancer surgery, it was shown that high AL values were associated with a 48% increased risk of postoperative complications, a 79% increased risk of an extended length of stay, and a twofold increase in the risk of mortality within 30 days of surgery. These data clearly indicate that taking AL into account before surgery may be key to increasing the chances of successful surgical treatment.

Although studies in breast, colorectal, and lung cancer consistently show the prognostic value of allostatic load, direct extrapolation to HNC requires caution. HNC patients face distinct challenges—tumor-induced dysphagia, aspiration, airway obstruction, poor nutrition, alcohol abuse, and functional/aesthetic deficits—that generate chronic stress and may uniquely amplify AL. Dedicated HNC studies are therefore essential.

It is worth noting that AL partially explains existing socioeconomic and racial disparities in cancer treatment outcomes. Patients with lower socioeconomic status and greater social vulnerability exhibit significantly higher AL values, which in turn are associated with poorer survival and greater toxicity from chemotherapy and radiotherapy [26].

While traditional AL panels focus on cardiovascular (blood pressure), metabolic (glucose, HbA1c, lipids), inflammatory (CRP, IL-6), and neuroendocrine (cortisol) biomarkers, nutritional status indicators are less commonly included. In HNC, however, parameters such as serum albumin, prealbumin, BMI, and lean body mass may serve as valuable adjuncts to AL assessment [27]. Malnutrition and cachexia—highly prevalent in HNC due to dysphagia, tumor-induced inflammation, and substance abuse—directly contribute to physiological ‘wear and tear’ and are associated with poorer postoperative outcomes. Future AL studies in the HNC population should consider integrating nutritional biomarkers into the composite score, as they may capture a disease-specific dimension of allostatic burden.

It appears that AL should be increasingly utilized in routine clinical practice in the coming years. Unfortunately, most studies focus on breast, colorectal, or lung cancers. Of course, given the prevalence of these cancers, this is justified; however, in this context, greater attention should also be paid to HNC.

### 3.3. Allostatic Load in Head and Neck Cancers—A Knowledge Gap

Despite the growing importance of AL in oncology, knowledge regarding its utility in HNC remains limited. This stems from the fact that HNC constitutes a specific group of cancers. In their course, classic risk factors such as smoking, alcohol abuse, and low socioeconomic status also contribute substantially to allostatic load. Additionally, they combine with poor nutrition and poor dentition, and patients are often individuals with addiction disorders. As a result, all these risk factors overlap and create a feedback loop between them, which ultimately makes it difficult to understand the precise role of AL in HNC and to accurately estimate it. This constitutes a knowledge gap, particularly when considering the prevalence of HNC and the fact that patients with these cancers typically have a high risk of developing depression and anxiety as early as the preoperative period [28]. This, combined with the aforementioned socioeconomic burden, may realistically translate into high AL levels in the preoperative period, which in turn—as demonstrated in patients with breast and colorectal cancer—may be associated with a higher risk of postoperative complications [5,11].

HNCs are characterized by a specific profile of risk factors and psychosocial burden. Patients in this group often have a history of long-term exposure to classic carcinogens, such as smoking or alcohol abuse, which contribute to the development of chronic inflammation and the activation of the HPA axis [6,7]. Added to this are low socioeconomic status, social isolation, stigma associated with tumor location, and significant functional (swallowing, speech, and breathing disorders) and aesthetic deficits, which dramatically impair quality of life as early as the diagnosis stage [8,9]. These factors contribute to a significant increase in AL.

To the best of our knowledge, evidence directly evaluating AL in HNC patients—particularly in relation to postoperative complications—remains lacking.

This knowledge gap warrants further research, both in terms of treatment decisions and quality of life. Introducing AL as a tool for preoperative risk stratification could identify patients who require prehabilitation, as their risk of postoperative complications—without such optimization—would otherwise be unacceptably high, as suggested by studies in other cancer populations [5,10,11]. From a scientific perspective, incorporating AL could help elucidate the mechanisms through which psychosocial and behavioral factors influence oncological outcomes in this population—a link already observed in other malignancies [3].

Filling this knowledge gap is therefore not only scientifically justified but also clinically urgent. Assessing AL in the HNC population could serve as a valuable component of precision medicine, enabling better prediction of treatment outcomes, optimization of multidisciplinary care, and reduction in disparities in access to effective therapy, as allostatic load has been shown to partially explain socioeconomic and racial differences in cancer outcomes [26]. Future studies should focus on the prospective assessment of AL prior to treatment, its dynamics during therapy, and its potential modifiability through interventions such as prehabilitation.

### 3.4. Prehabilitation and Its Potential Impact on Modifying Allostatic Load in HNC

The goal of prehabilitation is to improve the patient’s physical and mental condition and ensure adequate nutrition prior to major surgery. Prehabilitation is currently an essential component of preparing patients for oncological surgery [29]. In this context, prehabilitation aims to reduce the risk of postoperative complications, shorten the length of hospital stay, and improve quality of life and prognosis. In the context of HNC, where patients already face a significant disease burden, prehabilitation appears to be extremely valuable, as beyond improving health status prior to surgery, it may hypothetically also contribute to reducing AL.

Prehabilitation and enhanced recovery after surgery (ERAS) compliance, although sharing the common goal of reducing surgical stress and improving treatment outcomes, represent distinct yet complementary strategies. ERAS standardizes perioperative care by implementing uniform protocols throughout the surgical journey, whereas prehabilitation actively enhances the patient’s physical reserve, nutritional status, and psychological resilience prior to surgery [30]. Through this synergistic approach, multimodal prehabilitation not only optimizes but also substantially amplifies the beneficial effects of the ERAS pathway in patients with head and neck cancer.

Dysphagia and dyspnea represent significant challenges in patients with HNC. Dysphagia, resulting from tumor location, local invasion, and the side effects of radiochemotherapy, leads to impaired swallowing, aspiration, weight loss, and malnutrition [10]. In turn, dyspnea caused by airway obstruction—particularly in advanced tumors of the oral cavity, pharynx, and larynx—constitutes a life-threatening condition and frequently requires urgent tracheostomy [31]. Both symptoms substantially impair patients’ quality of life and increase the risk of postoperative complications. Therefore, prehabilitation focused on improving swallowing function, optimizing nutritional status, and enhancing respiratory performance plays a particularly important role in the comprehensive preoperative preparation of HNC patients.

Multimodal prehabilitation for HNC involves a wide range of interventions due to the specific nature of this patient group. It typically includes exercise training to improve physical fitness, individualized nutritional intervention, a high-protein diet, and psychological support. Furthermore, given the characteristics of HNC, specific elements such as cessation of substance use and speech-language therapy targeting swallowing and speech disorders are essential [32].

From an AL perspective, prehabilitation can benefit HNC patients in several ways [10]. First and foremost, physical activity—both aerobic and resistance training—has a significant effect on reducing inflammatory markers such as CRP, interleukin-6 (IL-6), and tumor necrosis factor alpha (TNF-α) [10]. In turn, nutritional interventions that improve nutritional status and body composition can positively affect glucose and lipid metabolism as well as blood pressure, thereby reducing metabolic and cardiovascular burden [10]. Perhaps the most important aspect in the context of HNC is psychological support (e.g., cognitive-behavioral therapy, relaxation techniques, or support groups)—it reduces chronic perceived stress, lowers cortisol and other stress hormones, which may directly reduce the neuroendocrine component of AL.

Data on the impact of prehabilitation on prognosis in HNC are limited; however, for other cancers, prehabilitation has been shown to have a positive effect on postoperative outcomes. The use of multimodal prehabilitation in patients with colorectal and breast cancer contributed to a reduction in AL, which correlated with a reduced risk of postoperative complications and higher survival rates [5,11,12]. In the context of HNC, studies have not been conducted on such large patient groups; therefore, their value should currently be considered limited [32]. Nevertheless, a meta-analysis indicates that prehabilitation significantly reduces the risk of serious postoperative complications, mortality, the incidence of dysphagia, and length of hospital stay [33]. Importantly, the combination of physical exercise with nutritional support and psychoeducation has proven particularly effective in reducing inflammatory markers and improving the immune response, which may indirectly contribute to lowering AL.

Furthermore, prehabilitation may play a role in preoperative risk stratification. Measuring AL before and after prehabilitation could serve as an objective biomarker of the intervention’s effectiveness—one that is more precise than individual parameters (e.g., hemoglobin level or BMI alone). Patients with initially high AL could be eligible for more intensive, individually tailored prehabilitation programs, which would allow for greater personalization of preparation for surgical treatment and potentially reduce existing socioeconomic disparities in treatment outcomes [12,26].

The implementation of prehabilitation into routine clinical practice for HNC faces significant barriers, such as the need for close multidisciplinary collaboration, limited reimbursement for the intervention, and low feasibility among patients with advanced-stage disease [10]. Nevertheless, a growing number of clinical studies suggest that prehabilitation may become the standard of care in this patient group.

In summary, multimodal prehabilitation offers a real opportunity to modify AL in patients with HNC. Introducing AL measurement as a tool to monitor its effectiveness could fill an existing research gap and reinforce the value of this intervention.

## 4. Conclusions

Despite the growing importance of AL in oncology, its role in HNCs remains virtually unexplored. This is a significant gap, as HNC patients, due to their characteristics (exposure to tobacco and alcohol, low socioeconomic status, social isolation, and psychological and functional burden), are particularly vulnerable to high AL even before treatment [6,7,8,9,28]. The lack of prospective studies prevents the stratification of complication risk and the personalization of care in this group.

Multimodal prehabilitation, combining physical training, nutritional support, and psychological interventions, has the potential to modify AL; however, no studies have yet been conducted that directly assess this relationship in patients with HNC [10]. Meta-analyses confirm the benefits of prehabilitation in HNC (reduction in mortality, complications, dysphagia, and length of hospital stay) [33], but none of them used AL as an endpoint.

In addition, incorporating AL assessment into routine practice can yield tangible economic benefits. Identifying patients with high AL prior to surgery would allow for targeted prehabilitation, which in turn could reduce the rate of complications, shorten hospital stays, and lower treatment costs for both the hospital and the patient (fewer follow-up visits, lower expenses for treating complications). Ultimately, the goal is to improve patients’ quality of life, and this legitimizes the use of AL as a clinical tool, not just a scientific one.

### 4.1. Clinical Implications

Routine assessment of AL in multidisciplinary HNC treatment teams could improve clinical decision-making, enable earlier intervention in patients with high AL, and contribute to better treatment outcomes and quality of life. Addressing this research gap in the HNC population is therefore not only a scientific challenge but also an urgent clinical and economic need.

### 4.2. Study Limitations

This narrative review has several important limitations that should be taken into account when interpreting the presented findings. First and foremost, due to the nature of a narrative review, no formal inclusion or exclusion criteria were applied to the studies, which may have introduced a risk of selective literature selection and subjectivity in the data synthesis process. Furthermore, the considerable heterogeneity in methods for calculating allostatic load—including popular approaches such as the high-risk quartile method, clinical cutoff values, and z-score standardization—makes it difficult to directly compare results across individual studies and different patient populations [3,14,17].

Another important limitation is the complete lack of original prospective studies evaluating the role of AL specifically in head and neck cancers. Consequently, most conclusions are based on indirect evidence derived primarily from studies conducted in patients with breast, colorectal, and lung cancer [3,4,5,23,24,25]. Although the biological mechanisms associated with AL are largely universal, extrapolating these findings to the HNC population requires caution due to the clinical, functional, and psychosocial specificities of this patient group.

Furthermore, interpreting the results is complicated by the fact that many HNC patients receive medications that may affect specific biomarkers included in the AL (e.g., analgesics, antidepressants, or glucocorticoids), which may mask the actual level of AL [20]. Finally, although multimodal prehabilitation appears to be a promising approach to modifying AL, its feasibility in patients with advanced HNC has not yet been fully established.

Despite these limitations, this review highlights a significant knowledge gap and underscores the need for dedicated studies in the HNC population to better understand the role of AL and the potential for modifying it through prehabilitation.

### 4.3. Bullet Points

○ Allostatic load (AL) is a multisystemic measure of the body’s physiological “wear and tear,” which in oncology correlates with poorer survival, a higher incidence of complications, and lower treatment tolerance.
○ In head and neck cancers (HNC), the role of AL remains largely unexplored, even though HNC patients exhibit many risk factors for high AL (tobacco, alcohol, low socioeconomic status, psychological burden).
○ Multimodal prehabilitation has the potential to reduce AL, but studies in the HNC population are lacking; incorporating AL into preoperative assessment could improve risk stratification, lower treatment costs, and reduce socioeconomic inequalities.
○ Prospective cohort and randomized controlled trials with standardized AL measurement are needed for AL to become a tool of precision medicine in HNC.

## Figures and Tables

**Table 1:** Comparison of the main methods for calculating AL.

Method	Scoring Method	Typical Number of Biomarkers	Main Advantages	Main Limitations
High-risk quartile	1 point for each biomarker in the high-risk quartile	8–15	High sensitivity to subclinical changes	Strong dependence on sample distribution; difficult to compare across populations
Clinical cut-off	Points based on established clinical thresholds	6–12	Easy interpretation and good comparability	Underestimates subclinical disorders
Z-score	Sum or mean of standardized (z-transformed) values	8–20	Uses full continuous data; no arbitrary cut-offs	Computationally more demanding; sensitive to skewed distributions
System-weighted indices	Different weights assigned to physiological systems	10–18	Accounts for clinical relevance of each system	Difficulty in determining appropriate weights
Item Response Theory	Weighted according to item difficulty and discrimination	Variable	Statistically sophisticated and precise	Computationally intensive; less intuitive
Machine learning approaches	Algorithm-based models	Variable	Can capture complex biomarker interactions	Lack of standardization; difficult to interpret

## Data Availability

No new data were created.

## Abbreviations

The following abbreviations are used in this manuscript:

AL	Allostatic load
BMI	Body mass index
CRP	C-reactive protein
HNC	Head and neck cancers
HNSCC	head and neck squamous cell carcinomas
HPA	Hypothalamic–pituitary–adrenal
IL	Interleukin
IRT	Item response theory
TNF-α	tumor necrosis factor alpha
